# Detection and Molecular Characterization of the SARS-CoV-2 Delta Variant and the Specific Immune Response in Companion Animals in Switzerland

**DOI:** 10.3390/v15010245

**Published:** 2023-01-15

**Authors:** Evelyn Kuhlmeier, Tatjana Chan, Cecilia Valenzuela Agüí, Barbara Willi, Aline Wolfensberger, Christian Beisel, Ivan Topolsky, Niko Beerenwinkel, Tanja Stadler, Swiss SARS-CoV-2 Sequencing Consortium, Sarah Jones, Grace Tyson, Margaret J. Hosie, Katja Reitt, Julia Hüttl, Marina L. Meli, Regina Hofmann-Lehmann

**Affiliations:** 1 Clinical Laboratory, Department of Clinical Diagnostics and Services, Center for Clinical Studies, Vetsuisse Faculty, University of Zurich, Winterthurerstrasse 260, 8057 Zurich, Switzerland; 2 Department of Biosystems Science and Engineering, ETH Zurich, Mattenstrasse 26, 4058 Basel, Switzerland; 3 SIB Swiss Institute of Bioinformatics, 4058 Basel, Switzerland; 4 Clinic for Small Animal Internal Medicine, Vetsuisse Faculty, University of Zurich, Winterthurerstrasse 260, 8057 Zurich, Switzerland; 5 Department of Infectious Diseases and Hospital Epidemiology, University Hospital Zurich, University of Zurich, Rämistrasse 100, 8091 Zurich, Switzerland; 6 School of Veterinary Medicine, College of Medical, Veterinary and Life Sciences, University of Glasgow, Bearsden Road, Glasgow G61 1QH, UK; 7 MRC-University of Glasgow Centre for Virus, College of Medical, Veterinary and Life Sciences, University of Glasgow, Bearsden Road, Glasgow G61 1QH, UK; 8 Center for Laboratory Medicine, Veterinary Diagnostic Services, Frohbergstrasse 3, 9001 St. Gallen, Switzerland

**Keywords:** SARS-CoV-2, Delta, variant of concern, AY.129, AY.43, AY.4, B.1.617.2, animal, next-generation sequencing, phylogenetic analysis, variant specific antibodies, One Health, viral adaptation

## Abstract

In human beings, there are five reported variants of concern of severe acute respiratory syndrome coronavirus type 2 (SARS-CoV-2). However, in contrast to human beings, descriptions of infections of animals with specific variants are still rare. The aim of this study is to systematically investigate SARS-CoV-2 infections in companion animals in close contact with SARS-CoV-2-positive owners (“COVID-19 households”) with a focus on the Delta variant. Samples, obtained from companion animals and their owners were analyzed using a real-time reverse transcriptase-polymerase chain reaction (RT-qPCR) and next-generation sequencing (NGS). Animals were also tested for antibodies and neutralizing activity against SARS-CoV-2. Eleven cats and three dogs in nine COVID-19-positive households were RT-qPCR and/or serologically positive for the SARS-CoV-2 Delta variant. For seven animals, the genetic sequence could be determined. The animals were infected by one of the pangolin lineages B.1.617.2, AY.4, AY.43 and AY.129 and between zero and three single-nucleotide polymorphisms (SNPs) were detected between the viral genomes of animals and their owners, indicating within-household transmission between animal and owner and in multi-pet households also between the animals. NGS data identified SNPs that occur at a higher frequency in the viral sequences of companion animals than in viral sequences of humans, as well as SNPs, which were exclusively found in the animals investigated in the current study and not in their owners. In conclusion, our study is the first to describe the SARS-CoV-2 Delta variant transmission to animals in Switzerland and provides the first-ever description of Delta-variant pangolin lineages AY.129 and AY.4 in animals. Our results reinforce the need of a One Health approach in the monitoring of SARS-CoV-2 in animals.

## 1. Introduction

Severe acute respiratory syndrome coronavirus type 2 (SARS-CoV-2), which emerged in December 2019 in Wuhan, China, was responsible for more than 632 million confirmed cases in humans by 17 November 2022 and caused coronavirus disease 2019 (COVID-19) [1].

In the last twenty years, three new highly pathogenic human respiratory coronaviruses have emerged and led to global infections named severe acute respiratory syndrome coronavirus (SARS-CoV), Middle-East respiratory coronavirus (MERS-CoV), and SARS-CoV-2 [2]. They are all thought to have emerged via viral spillover from an animal reservoir into humans. The spillback of SARS-CoV-2 from humans to companion and wild animals has been documented in the literature [3,4,5,6,7,8,9,10,11,12,13,14,15].

Almost three years ago, the World Health Organization (WHO) declared a SARS-CoV-2 pandemic (March 2020) [16]. Since then, new SARS-CoV-2 variants with altered pathogenic characteristics appeared and were named as variants of concern (VOCs). The criteria for VOCs are an increase in transmissibility or harmful changes in COVID-19 epidemiology, an increase in virulence or change in clinical presentation, or a decrease in the effectiveness of public health measures or available diagnostics, vaccines, and therapeutics. Five VOCs have been documented to date: Alpha or B.1.1.7, which was first detected in the United Kingdom; Beta or B.1.351 in South Africa; Gamma or P.1 in Brazil; Delta or B.1.617.2 in India; and Omicron or B.1.1.529, which appeared in multiple countries in November 2021 [17].

Throughout the pandemic, 695 SARS-CoV-2 outbreaks in animals, affecting animals in 36 countries and infecting 26 different animal species, have been documented by the World Organisation for Animal Health (WOAH, founded as International Office of Epizootics (OIE; latest update: 31 November 2022) [18]. The first SARS-CoV-2-positive dogs from COVID-19-affected households in Hong Kong were reported early on during the pandemic [19]. Since then, natural SARS-CoV-2 infections in companion animals have been reported in several countries, including Switzerland [6], Italy and Germany [20], France [21], the United States of America (USA) [22,23], Spain [24,25], Portugal [26], Poland [27], Iran [28], and the United Kingdom (UK) [8]. Additionally, a number of SARS-CoV-2 variants have been documented in companion animals. The Alpha variant was found in cats and dogs in Italy [29], Argentina [30], Thailand [31], Spain [32], the USA [33], Germany [34], and the UK [35]. Delta infections have been described in cats [25] and dogs [36] in Spain, the USA [37], and China [38]. There are also a few reports regarding the AY.3 Delta pangolin lineage in cats and dogs in the USA [39,40].

The first SARS-CoV-2 molecularly and serologically confirmed positive cat in Switzerland was described in November/December 2020 by our group [6]. The cat had been infected with the SARS-CoV-2 lineage B.1.1.39, which was highly prevalent in Switzerland at that time. Subsequently, we initiated a prospective study with the goal to systematically investigate SARS-CoV-2 infections in companion animals living in contact with confirmed SARS-CoV-2-positive owners in COVID-19-affected households. Within that study, we aimed to determine the SARS-CoV-2 prevalence in animals in Swiss COVID-19 households and to assess the potential risk factors for infection. Moreover, we molecularly characterized the virus variants in animals and their owners using next-generation sequencing (NGS) and characterized the immune response in the companion animals. An overview of all the results and statistical analyses thereof, covering the time period of 11 June 2020 until 20 May 2022, will be presented elsewhere. In the present study, we include nine COVID-19 households recruited within the above-mentioned prospective study, which all harbor the SARS-CoV-2 Delta variant. The study aims to provide detailed information for each individual household, its humans and animals, the molecular and serological as well as genomic and phylogenetic analyses of the viruses found in each household, and an overall analysis of infection chronologies, transmission probabilities, as well as the role of surfaces in infection events. It is the first report of the SARS-CoV-2 Delta variant in Swiss companion animals (eleven cats and three dogs) and, to the best of our knowledge, also the first description in the world of the AY.4 and AY.129 Delta pangolin lineages in animals. Our results support the susceptibility of companion animals for different variants and subtypes and the importance of monitoring SARS-CoV-2 in companion animals as a One Health issue.

## 2. Materials and Methods

### 2.1. Study Design and Recruitment of COVID-19-Affected Households

The inclusion criteria for this study, for which samples were collected from 11 June 2020 until 20 May 2022, were RT-PCR-confirmed SARS-CoV-2 infections in at least one person of the household, independent of the health status of this person (“COVID-19-affected household”), and the presence of at least one companion animal in the household. Recruitment was achieved via information leaflets circulated in Switzerland, e.g., posters/flyers at the Vetsuisse Faculty Zurich, University of Zurich, and University Hospital Zurich, and also through the support of the cantonal medical and veterinary physicians as well as via contact tracing conducted by the veterinary office of the canton of Zurich. An informed consent sheet was provided. Once pet-owner consent was obtained, an individual sampling kit for each COVID-19-affected household was prepared. The sampling kit included the sample materials, personal protective equipment to prevent contamination, and detailed instructions. An email and telephone service for the owner was provided in case of concerns regarding the sampling procedure. Written consent was obtained from all participating owners. The study was approved by the ethics committee of the canton of Zurich (BASEC number 2020-00979) and the veterinary office of the canton of Zurich (ZH062/20).

### 2.2. Swab and Blood Sample Collection

A sampling kit containing 1.5 mL screw-lid tubes (Sarstedt AG and Co. KG, Nümbrecht, Germany), prefilled with 300 µL of DNA/RNA shield solution (Zymo Research Europe GmbH, Freiburg, Germany) as previously described [41], cotton swabs (Heinz Herenz, Hamburg, Germany), cytobrushes (Cepillo Cervical Cell Sampler, Deltalab, S.L., Rubi, Spain), disposable gloves (Nitrile gloves, SATRA Technology Europe Ltd., Dublin, Ireland), disposable surgical masks (Zhejiang Longde Pharmaceuticals Co., Ltd., Hangzhou, China), a detailed description of sampling, and return envelopes were provided to obtain each sample. Every sampling kit was packed under strict hygienic measures and all materials were separately placed in sealed, resealable plastic bags (Minigrip^®^ Red line, Alpharetta, GA, USA) to avoid any contamination. A disposable mask and gloves were provided in each sampling kit for the owners to wear whilst sampling their animals, so that the sampler would not contaminate the kit.

Swab samples were collected and analyzed to determine the presence of acute SARS-CoV-2 infection in the animal and their owners. For biosafety reasons, all animal and owner swab samples were collected by the owners. Three swab samples were obtained from each animal (oral, nasal, and fecal), one swab from the animal’s fur and one swab from the animal’s bed; moreover, a pillow sample was obtained from the bed of the SARS-CoV-2-positive person. These surface samples (fur, bed, and pillow) help us to learn about the magnitude and distribution of environmental contamination.

For this purpose, we investigated how often and for how long viral material can be detected on certain surfaces. Additionally, the SARS-CoV-2-positive person/s could voluntarily provide an oropharyngeal and nasal swab for a sequence comparison with the animal to investigate phylogenetic similarity of the SARS-CoV-2 strains.

The swab samples obtained from all COVID-19-affected households were collected between 16 November 2021 and 13 January 2022. We aimed to collect swab samples from positive companion animals every other day until the animal tested negative using reverse transcriptase-polymerase chain reaction (RT-qPCR). Moreover, a blood draw was planned once the animal tested negative after confirmed SARS-CoV-2 infection, either at the University Animal Hospital Zurich or at their local veterinarian. From animals that tested RT-qPCR-negative in COVID-19-affected households, the swabs were obtained at two follow-up timepoints, each one week apart.

### 2.3. SARS-CoV-2 Nucleic Acid Extraction, Analysis, and Confirmation

The screw-lid tubes were unpacked in a laminar flow cabinet and rinsed with 70% ethanol, wiped, and subsequently put on a shaking incubator at 42 °C for 10 min at 600 rpm. After the incubation, all tubes were centrifugated and the swabs were inverted and removed as previously described [6,42,43,44].

Total nucleic acid/ribonucleic acid (TNA/RNA) extraction of the swab samples was performed with the MagNA Pure 96 instrument (Roche Diagnostics AG, Rotkreuz, Switzerland) using the Viral NA Plasma ext lys SV protocol, and the MagNA Pure 96 DNA and Viral NA Small Volume Kit (Roche Diagnostics AG) or the QIAamp RNA blood mini kit (Qiagen, Hilden, Germany), following the manufacturers’ instructions.

SARS-CoV-2 analysis was conducted on an ABI PRISM 7500 Fast Sequence Detection System (Applied Biosystems Foster City, CA, USA). Two SARS-CoV-2 real-time RT-qPCR assays were used (envelope gene sequence (E) and the RNA dependent-RNA polymerase sequence (RdRp), as described in Klaus et al., 2021 [6]).

RT-qPCR-positive animal swab samples (oral, nasal, and fecal) were sent to the Swiss Federal Institute of Virology and Immunology (IVI; Mittelhäusern, Switzerland), the veterinary reference laboratory of Switzerland, for confirmation of the SARS-CoV-2 result. Samples were judged as “positive” if in both assays the cycle threshold (Ct) values were ≤38, “questionable positive” if the Ct values were >38 and <45, and “negative” if Ct values were ≥45, as previously described [20]. Confirmed RT-qPCR-positive animals were reported by the Federal Food Safety and Veterinary Office to the WOAH.

### 2.4. Next-Generation Sequencing (NGS) and Phylogenetic Tree Construction

NGS was conducted by the Genomics Facility Basel, Eidgenössische Technische Hochschule Zürich (ETH), Basel, Switzerland. For this purpose, extracted TNA/RNA from RT-qPCR-positive animal swab samples was sent to the Genomics Facility Basel.

Library preparation and whole-genome sequencing was performed on Illumina MiSeq and NovaSeq 6000 NGS systems, as previously described by Nadeau et al. [45].

For the quality control and processing of raw sequencing reads, the bioinformatic pipeline V-pipe [46], with the SARS-CoV-2 base configuration, was used. Consensus sequences with <20,000 bases called and with >15 excess mutations were rejected. To control for potential contamination due to the low viral load of the samples, several negative controls were included in the sequencing and analyzed with the same bioinformatic pipeline. Genome positions identified as prone to contamination according to the negative controls were masked in the following genetic analyses. The whole-genome consensus sequences were submitted to the Global Initiative on Sharing All Influenza Data (GISAID) [47]. Lineage assignment was performed with the pangolin tool (Pango v.4.1.3 constellation v0.1.10) [48].

For the phylogenetic analysis, the Nextstrain ncov pipeline (GitHub—nextstrain/ncov; https://github.com/nextstrain/ncov/; accessed on 9 May 2022) was used [49]. Sequences with a length > 27,000 and complete date from October 2021 to January 2022 were downloaded from GISAID on 15 May 2022. Sequences were pairwise aligned against the Wuhan reference sequence (GenBank MN908947.3) with Nextalign, a variation of the Smith–Waterman algorithm [49].

Based on the sequence alignment, we reconstructed phylogenetic trees. Following the Nextstrain pipeline, sites prone to low sequencing accuracy were masked prior to the phylogenetic inference: sites 21846, 21987, 22992, 23012, 23063, 23604, and 24410, as well as 100 sites at the beginning and 200 at the end of the sequences. The maximum-likelihood tree was estimated with IQTREE2 [50] under the GTR substitution model with 10,000 bootstrap replicates [51]. To root the tree, the reference genome Wuhan-Hu-1/2019 (GenBank: MN908947) was used as an outgroup.

We reconstructed a phylogenetic tree including all the animal and owner viral sequences in the current study from an alignment of 348 sequences. From these 348 sequences, 7 were obtained from animal samples in the study, 12 were from the owner samples in the study, 41 were all high-coverage sequences assigned as Delta variants from dogs and cats submitted to GISAID from other countries, 241 sequences were randomly selected from all the sequences from the canton of Zurich, 46 sequences were randomly selected from any country, and 1 was the reference sequence Wuhan/Hu-1/2019. All sequences were collected from November 2021 to January 2022. Based on the maximum-likelihood tree, a time-scaled phylogeny was estimated with TreeTime [52] and a clock rate of 0.0008 substitutions/site/year [49].

Further, we reconstructed one household-focused phylogenetic tree for each COVID-19-affected sequenced household: USZ22, USZ29, USZ36, USZ38, and USZ39. For each household-focus tree, an alignment with the household sequences, the 10 most genetically similar sequences to each of the household sequences and a random subset of 10 sequences (context sequences) from the same canton, pangolin lineage, and collection date (+/−15 days) of the household samples was reconstructed. Genetic similarity was calculated using Nextstrain priority protocol based on pairwise SNP distance.

Single-nucleotide polymorphism (SNP) figures were generated with snipit (GitHub—aineniamh/snipit; https://github.com/aineniamh/snipit: accessed on 10 May 2022). We used cov-spectrum/LAPIS [53,54] to obtain the proportions of the different SNPs and lineages with respect to all the sequences obtained from Switzerland uploaded to GISAID. We considered the distance between two viral sequences as the number of different SNPs between them, without accounting for missing nucleotides.

### 2.5. Serological SARS-CoV-2 Testing and Variant Analysis

The animal sera were tested using an in-house-developed enzyme-linked immunosorbent assay (ELISA) that detects antibodies binding to the SARS-CoV-2 spike glycoprotein receptor-binding domain (RBD), as described [6] by the following modifications. As an antigen, SARS-CoV-2 spike protein RBD, Strep-Polyhistidine-tagged (LU2020-1MG, LubioScience, Zurich, Switzerland) was used. Therefore, the threshold for suspicious samples was also changed from the previous optical density (OD) value cut-off (cats: >0.5; dogs: > 0.3) to a mean concentration (MC) cut-off (cats: MC+1x standard deviation (SD); dogs: MC+3xSD) for standardization purposes.

A rabbit anti-dog immunoglobulin (IgG) horseradish-peroxidase (HRP)-conjugated secondary antibody (Jackson ImmunoResearch Europe, Ely, UK) was used for dog samples.

As a positive control for the cat samples, a previously positive-tested cat (25607; collected on 15 March 2021 for another project about COVID-19) and domestic animals (TVB: ZH062/20) was used; for the dogs, three positive-tested samples from our laboratory (USZ6 Animals 4 and 5, confirmed positive in Utrecht by S1/RBD ELISA and VN; confirmed positive in Glasgow by PVNT) were used and mixed in equal proportions.

The negative control for the cat samples was a pool of specified pathogen-free cats (JJF1/JJG3/JJG4/JJI1) collected in 2017 [55]. For the dog samples, a sample tested negative via the RBD ELISA and SARS-CoV-2 Surrogate Virus Neutralization Test Kit (sVNT) in our laboratory and confirmed negative via pseudotype-based neutralization assay (PVNT) in Glasgow (USZ234 Animal 1) was used. The 30 Swiss cat sera, which had been collected for a previous unrelated project between 12 April 2019 and 26 July 2019, were used as pre-SARS-CoV-2 samples. The pre-SARS-CoV-2 samples of the dogs consisted of 27 samples from 2017, obtained from a previous study [56].

Additionally, serum samples were tested with a commercially available SARS-CoV-2 Surrogate Virus Neutralization Test Kit (sVNT; GenScript Inc., Piscataway, NJ, USA) for the detection of neutralizing activity against SARS-CoV-2 RBD of the spike protein of the antibodies already detected with RBD ELISA. The test was performed according to the manufacturer’s protocol and as previously described (cut-off: MC+6xSD) [20].

Finally, the neutralizing antibody activity of the serum samples was measured using a pseudotype-based neutralization assay (PVNT). The human immunodeficiency virus (SARS-CoV-2) pseudotypes bore the spike protein of one of four SARS-CoV-2 variants: B.1 (Wuhan D614G), B.1.1.7 (Alpha), B.1.617.2 (Delta), or B.1.1.529 (Omicron BA.1). Samples were tested against the four pseudotypes within the same assay and the titers compared. Samples with no measurable titers were considered negative. For samples with one or more titers, the pseudotype variant that generated the highest titer indicated the variant the animal had likely been infected with. The assay was performed as previously described by Davis et al. [57,58]. For this study, the SARS-CoV-2 spike glycoprotein expression constructs were synthesized using GenScript (Netherlands). Constructs bore the following mutations relative to the Wuhan-Hu-1 sequence (GenBank: MN908947): B.1 (Wuhan D614G)—D614G; B.1.1.7 (Alpha)—Δ69-70 (69-70-deletion), Δ144, N501Y, A570D, D614G, P681H, T716I, S982A, D1118H; B.1.617.2 (Delta)—T19R, G142D, Δ156-157, R158G, L452R, T478K, D614G, P681R, D950N. B.1.1.529 (Omicron BA.1)—A67V, Δ69-70, T95I, G142D/Δ143-145, Δ211/L212I, ins214EPE, G339D, S371L, S373P, S375F, K417N, N440K, G446S, S477N, T478K, E484A, Q493R, G496S, Q498R, N501Y, Y505H, T547K, D614G, H655Y, N679K, P681H, N764K, D796Y, N856K, Q954H, N969K, L981F. All synthesized S genes were codon-optimized, incorporated the mutation K1255STOP to enhance surface expression, and were cloned into the pcDNA3.1(+) eukaryotic expression vector.

## 3. Results

### 3.1. Overview of COVID-19-Affected Households Included in This Study, Characterization of Cats and Dogs and Available Material, and Test Results

Households: nine COVID-19-affected households were included in the study. They housed a total of eighteen companion animals, fifteen cats (samples available from fourteen cats), and three dogs. All three dogs and eleven of fourteen tested cats were positive for the SARS-CoV-2 Delta variant (molecularly or serologically; Table 1). Households were located in the cantons of Zurich and St. Gallen. The households in which the delta variant antibodies were identified, tested positive between mid-November 2021 to mid-February 2022.

Cats: swab samples for RT-qPCR were available from 11 cats. Seven of the eleven cats tested positive for SARS-CoV-2 RT-qPCR, and an eighth cat (49.1) was a questionable positive using a RT-qPCR (Table 1). Moreover, serum samples were available from nine cats. This included six cats that had already tested positive for RT-qPCR and three additional cats (174.1, 174.2, and 211.1) that tested serologically positive for SARS-CoV-2 infection (Table 1). In four cats from three COVID-19-affected households, clinical signs (respiratory and/or gastrointestinal) were reported by the owner (Table 2). In three of these four cats, SARS-CoV-2 infection was confirmed in this study; for the fourth cat (38.1), only swab samples (three oral, three nasal) were available, which tested negative using a RT-qPCR; no serum sample was available to check serologically for infection. A description of the different cats (age, sex, and breed), pre-existing conditions, and observed clinical signs are presented in Table 2.

Dogs: swab samples were available for RT-qPCR from two of the three dogs: both tested SARS-CoV-2-positive using a RT-qPCR. Moreover, serum samples were available from all three dogs (22.1, 29.1, and 149.1), and for all of them, evidence of infection could be detected serologically (Table 1). In two dogs (29.1 and 149.1), clinical signs were reported (Table 3).

Swab sample collections in the households started with an average interval between the first positive test from the owner and the first animal sample of 5.2 days (range 2 to 9 days). Samples were collected at three to nine timepoints within a period of 14 to 29 days (mean: 18.2 days).

Most of the positive cats (91%; 10/11) and 100% (3/3) of the dogs were found to be infected with the SARS-CoV-2 Delta variant as determined either via NGS and/or variant-specific serology (Table 1, details see below). For one cat (29.3), no NGS data or material for serology were available.

### 3.2. Timeline of Sample Collection and Molecular and Serological Testing for SARS-CoV-2 in the Different Households

**Figure 1:**
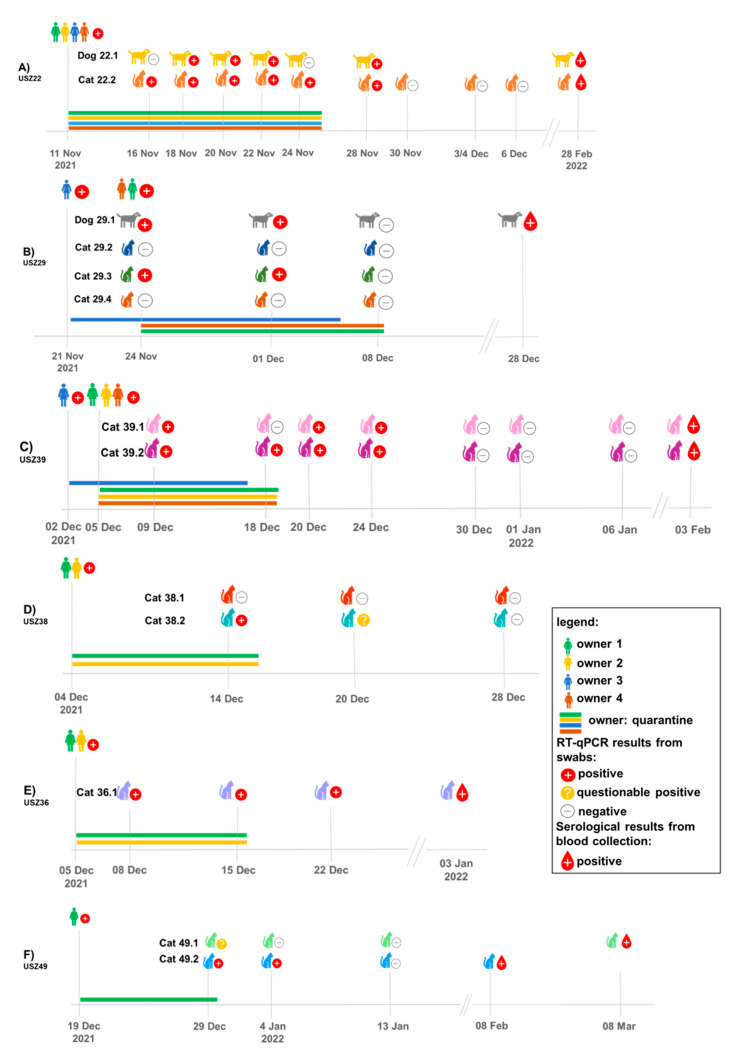
Overview of testing and results (RT-qPCR and serology samples) of animals and owners in households USZ22 (**A**); USZ29 (**B**); USZ39 (**C**); USZ38 (**D**); USZ36 (**E**); and USZ49 (**F**). Only owners that tested positive are shown. All households shown are located in the canton of Zurich.

Details of all nine households included in this study and the timeline of sampling as well as positivity using a RT-qPCR and serology are presented in chronological order of study entry in Figure 1 and further details on all the animals used in this study (age, breed, sex, and pre-existing conditions) are presented in Table 2 for cats and Table 3 for dogs.

Subsequently to the first infection, the household USZ22 reported a second RT-qPCR-confirmed SARS-CoV-2 infection of three owners with Omicron (BA1.1) in January 2022, and at that time participated in the study for a second time. However, all oral, nasal, and fecal swab samples obtained from dog 22.1 and cat 22.2 were RT-qPCR-negative at that time (22 January 2022 to 3 February 2022). The two animals did not show any neutralizing activity to the Omicron variant in the blood sample collected on 28 February 2022 while both animals tested serologically positive for the Delta variant, 104 days after they had tested positive for the first time via RT-qPCR for the Delta variant.

Three additional households from which only blood samples were available were also included: Household USZ174 with two adults, who tested SARS-CoV-2-positive on 11 December 2021, was located in the canton of Zurich (Table 1) and housed two cats (174.1 and 174.2) (Table 2). No swab samples were available from this household, but blood samples obtained from the two cats were collected on 21 December 2021 at the Small Animal Clinic of the Vetsuisse Faculty Zurich, Switzerland. Both cats tested serologically positive at that time, 10 days after the infection had been confirmed in the owners (household not included in Figure 1 since only one timepoint was sampled).

Household USZ149 (actually a dog kennel) from the canton of St. Gallen was enrolled in the study on 21 December 2021 through the attending veterinarian in St. Gallen (Table 1), where the dog (149.1) had been presented with sneezing. The dog had been housed in a dog kennel in August 2021, where one of the animal caretakers had been SARS-CoV-2-positive at that time. A blood sample was obtained from the dog upon presentation at the attending veterinarian on 21 December 2021. At that time (approximately 120 days after the animal caretaker was reported to be positive), the dog tested serologically positive for SARS-CoV-2. No swab samples were available from this dog or the animal caretaker. Households USZ149 and USZ211 are not included in Figure 1 because of limited sampling timepoints.

Household USZ211 (not included in Figure 1) was enrolled on 9 February 2022 when cat 211.1 had been presented with gastrointestinal signs to a veterinarian in St. Gallen while the owner of the cat had been infected with SARS-CoV-2 (Table 1). No swab samples were available from cat 211.1, but the cat tested serologically SARS-CoV-2 positive at that time. Samples were not available from a second cat or from the owner living in the household.

### 3.3. Detection of SARS-CoV-2 Viral RNA and RNA Loads in Different Samples from Companion Animals and the Environment (Fur, Bedding)

**Figure 2:**
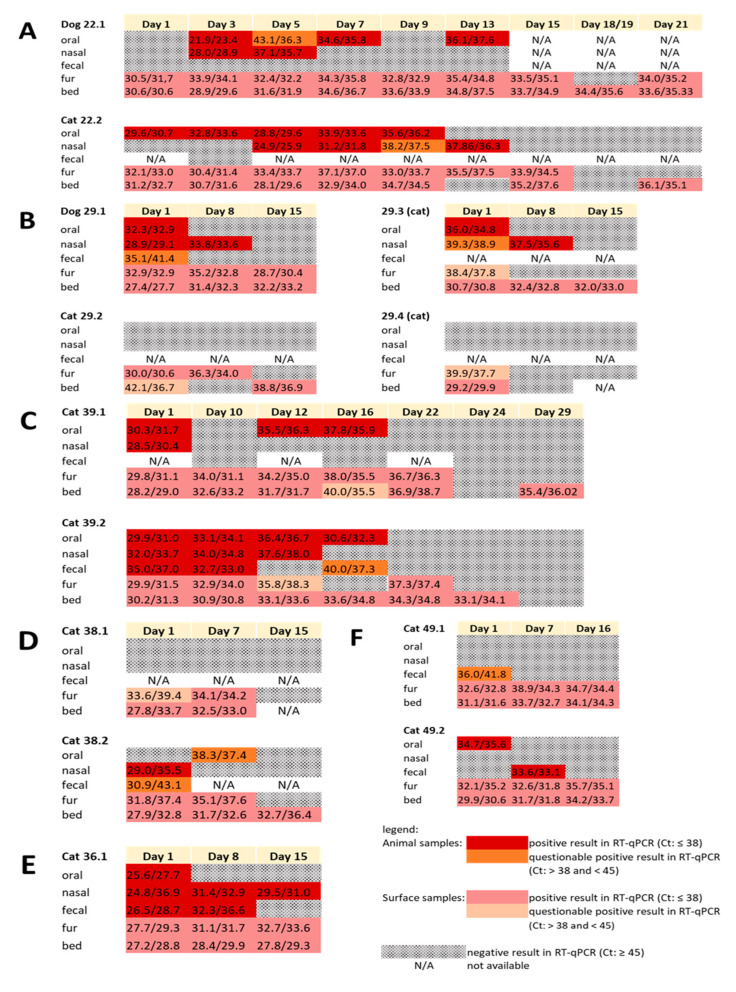
Real-time reverse transcriptase polymerase chain reaction (RT-qPCR) results of households USZ22 (**A**); USZ29 (**B**); USZ39 (**C**); USZ38 (**D**); USZ36 (**E**); and USZ49 (**F**). Results of the different tested assays (E/R assay), collection timepoints (initial and follow-ups), and different sample materials (oral, nasal, fecal, fur, and bed) are depicted.

In household USZ22, oral and nasal samples obtained from dog 22.1 and cat 22.2 tested RT-qPCR-positive at different timepoints, while fecal samples from dog 22.1 were negative and from cat 22.2 only one fecal sample was available, which was negative (Figure 2A). The highest viral load value for the dog was found in an oral sample (Ct value 21.9) and for the cat in a nasal sample (Ct value 24.9). Surface samples (fur and bed) tested positive over the entire sampling period with only few exceptions, and with that they tested positive at least eight days longer than the animal samples (Figure 2A).

In household USZ29, dog 29.1 and cat 29.3 tested SARS-CoV-2-positive in oral and nasal swabs at different timepoints, and the dog tested questionably positive for the first fecal swab (Figure 2B). The household has reported, that the children had less contact with cats 29.2 and 29.4, which did not test positive at any of the timepoints tested. Fur and bed samples tested positive not only for the positive-tested dog 29.1 and cat 29.3, but also for the two RT-qPCR-negative-tested cats 29.2 and 29.4 (Figure 2B). Only a few samples had a Ct value below 30.

In household USZ39, in both SARS-CoV-2 RT-qPCR-positive cats (39.1 and 39.2), oral and nasal swabs tested positive, while only in one cat (39.2) the fecal swabs were also found to be positive (Figure 2C). The fur and cat bed swabs were positive for longer than three weeks, and with that up to 13 days longer than the cats tested RT-qPCR-positive. Only some samples from the first test timepoint had a Ct value slightly lower than 30.

In household USZ38, only one nasal sample from cat 38.2 tested positive and an oral and a fecal sample tested questionably positive, while fur and cat bed samples tested positive from both cats 38.1 and 38.2 (Figure 2D). Highest loads were observed in the surface and not in animal samples (Ct values around 28).

In household USZ36, initially, oral, nasal, and fecal samples tested positive by RT-qPCR; the fecal sample remained positive after one week and the nasal sample also at the last sampling timepoint after two weeks (Figure 2E). In this cat, initial viral RNA loads were similar in all three samples, oral, nasal, and fecal, with Ct values of 24.8 to 26.5. Fur and bed samples were positive at all three collection timepoints (testing period 15 days), and the viral loads were similarly high in the surface samples as in the animal samples.

In household USZ49, cat 49.1 tested questionably positive in the first fecal swab, was but otherwise negative, while cat 49.2 was positive in one oral and one fecal sample (Figure 2F). In contrast, the surface samples (fur and bed) obtained from both cats tested positive over the entire sampling period and with higher loads (lower Ct values) than the animal samples (Figure 2F). However, only one sample from bedding had a Ct value slightly lower than 30.

Overall, a large proportion of fur (72.9%; 43/59) and animal bed samples (89.5%; 51/57) tested RT-qPCR-positive for SARS-CoV-2 RNA (Figure 2A–F), while only 33.6% (48/143) of the animal samples tested positive. The surface samples tested RT-qPCR-positive up to 13 days after the last positive test of the animals with the lowest Ct values of 29 for fur and 28 for animal bed samples.

### 3.4. Confirmation of SARS-CoV-2 Infection in Cats and Dogs by Serology

From 12 animals (three dogs and nine cats) in eight COVID-19-affected households’ blood samples were available for the serological confirmation/detection of SARS-CoV-2 infection (Table 1). In eight of these 12 animals, SARS-CoV-2 infection had already been detected using RT-qPCR (two dogs and six cats) (Table 1). In seven out of these eight animals, with the exception of dog 22.1, an infection could be confirmed by RBD ELISA or the sVNT test (Table 4). In all these seropositive-tested animals, the greatest neutralizing activity in PVNT was observed for the SARS-CoV-2 Delta variant (Table 5). In dog 22.1, which was seronegative according to RBD ELISA and sVNT, a low-positive PVNT titer of 58 against the Delta variant was detectable (Table 5). Moreover, there were four animals (one dog and three cats) lacking swab samples and thus RT-qPCR results. All these animals entered the study because of positive RBD ELISA and sVNT results (Table 4). In all four animals, the highest titer was observed against the Delta variant of SARS-CoV-2 (Table 5).

### 3.5. Confirmation of SARS-CoV-2 Delta Variant Infection and Determination of the Pangolin Lineage in Cats, Dogs, and Owners by NGS

The nearly complete SARS-CoV-2 viral genome was successfully sequenced for 19 samples from five cats, two dogs, and 12 companion animal owners (Table 6) with high coverage, >20,000 bases and with fewer than 15 excess mutations not observed in other sequences. One owner and one cat sample revealed insufficient coverage of the sequence and was excluded from further analysis (Table 6).

All samples obtained from the same household, owner, and animal were infected by the same pangolin lineage. A cat (22.2) and a dog (22.1) from one household (USZ22) were infected with the pangolin lineage AY.129, two cats (39.1, 39.2) from one household (USZ39) were infected with pangolin lineage B.1.617.2, one cat (36.1) was infected with pangolin lineage AY.43, and a dog (29.1) and cat (38.2) from two different households (USZ29 and USZ38) were infected with pangolin lineage AY.4 (Table 6).

**Figure 3:**
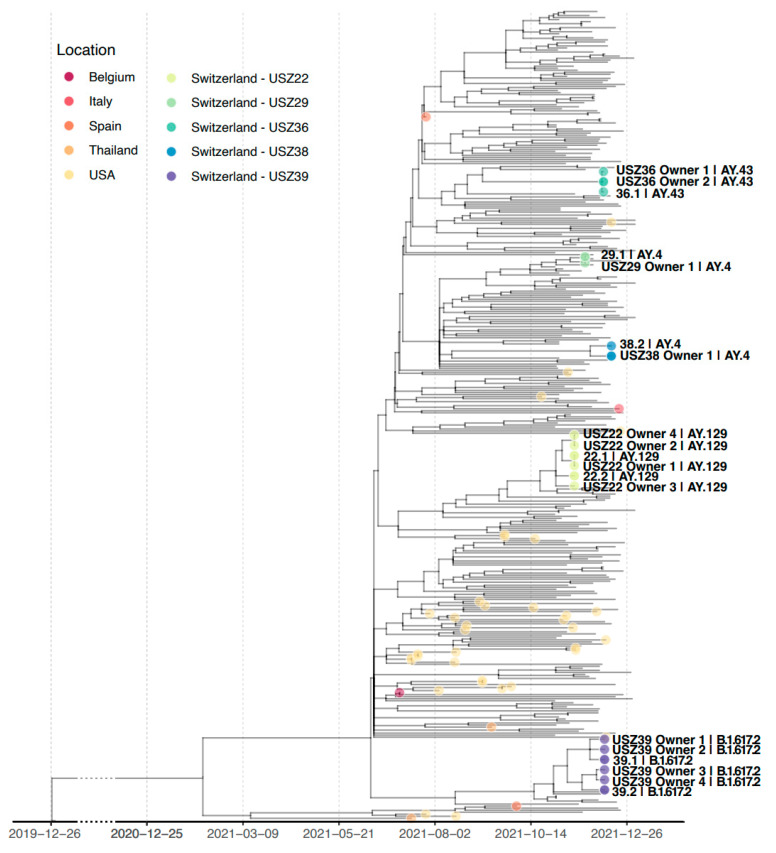
Time-scaled maximum-likelihood phylogenetic tree with viral sequences from companion animals and owners of households USZ22, USZ29, USZ36, USZ38, and USZ39. A total of 330 sequences are included to represent the genetic diversity of the SARS-CoV-2 Delta variant in Zurich from November 2021 to January 2022 and the genetic diversity of the SARS-CoV-2 Delta variant in companion animals worldwide (see Materials and Methods Section for a detailed description of the alignment). Sequences from the study and companion animals are indicated with a dot in the tree; only sequences from the study are labeled with the sample name and pangolin lineage. Tip color indicates the location of the sequence. Time scale is shown at the bottom of the figure; a clock rate of 0.0008 substitutions/site/year is assumed for the reconstruction of the time-scaled tree. Branch length in the tree represents evolutionary distance, i.e., substitutions per site. Reference sequence Wuhan/Hu-1/2019 is used as an outgroup to root the tree.

**Figure A1:**
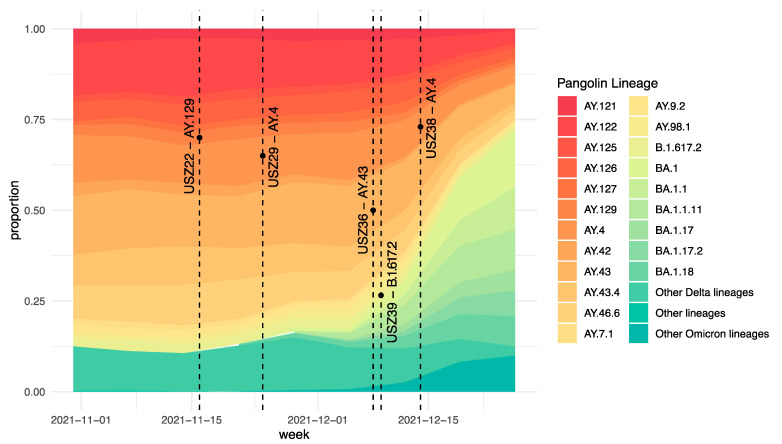
SARS-CoV-2 lineage distribution of Zurich during the sampling period. This plot shows each household and lineage (black dots) of the study and the time of sampling (dashed lines).

When constructing a phylogenetic tree including all sequences obtained within this study, sequences from each household were grouped in a separate cluster (Figure 3). The proportion of cases in the canton of Zurich per identified pangolin lineage at the corresponding point in time is also visible in Figure A1.

The NGS results are discussed according to the lineage identified in the households: first AY.129 (USZ22), then AY.4 (USZ29 and USZ38), AY.43 (USZ36), and finally B.1.617.2 (USZ39).

In household USZ22, both companion animals, dog 22.1 and cat 22.2, as well as the four owners were infected with the Delta variant AY.129 (Nextclade clade 21J). AY.129 (alias for B.1617.2.129) was a European lineage and the proportion in the canton of Zurich in the calendar week 46; the timepoint the owners tested positive for the first time was 7.2% of all sequenced samples in the canton of Zurich [53,59].

**Figure 4:**
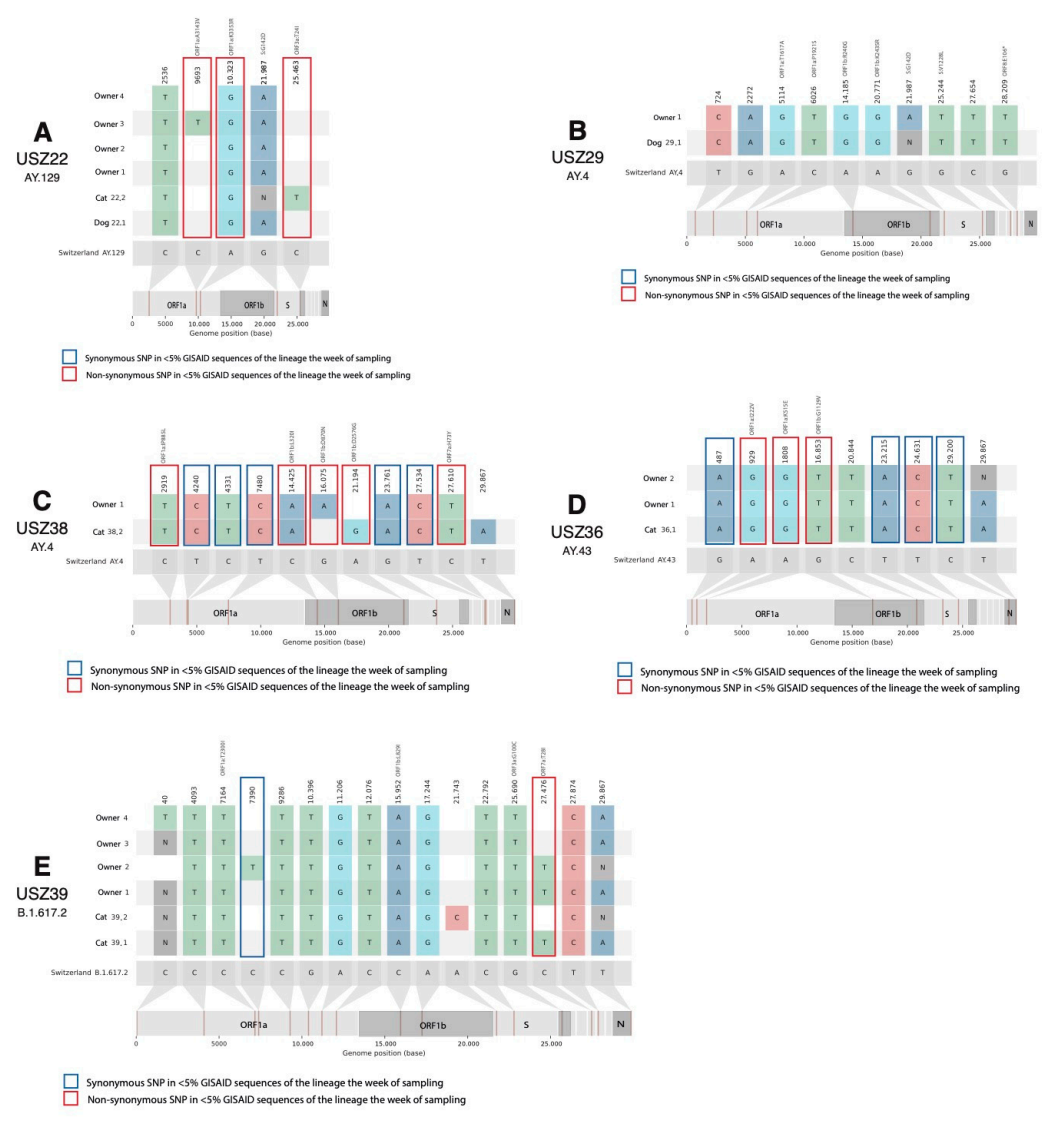
Snipit plot with nucleotide mutations for the five identified households with NGS data. Only mutated positions that are not common for the designated lineage (<75% GISAID sequences of the lineage) are shown. Mutations that are shared by less than 5% of the identified lineage (AY.129 for **A**, AY.4 for **B**,**C**, AY.43 for **D**, and B.1.617.2 for **E**) sequences in Switzerland in the week of sampling are indicated with a box: red for non-synonymous SNPs and blue for synonymous SNPs. (**A**) household USZ22, (**B**) household USZ29, (**C**) household USZ38, (**D**) household USZ36, and (**E**) household USZ39. Amino acid substitutions are indicated in gray on top of the non-synonymous mutations.

**Figure A2:**
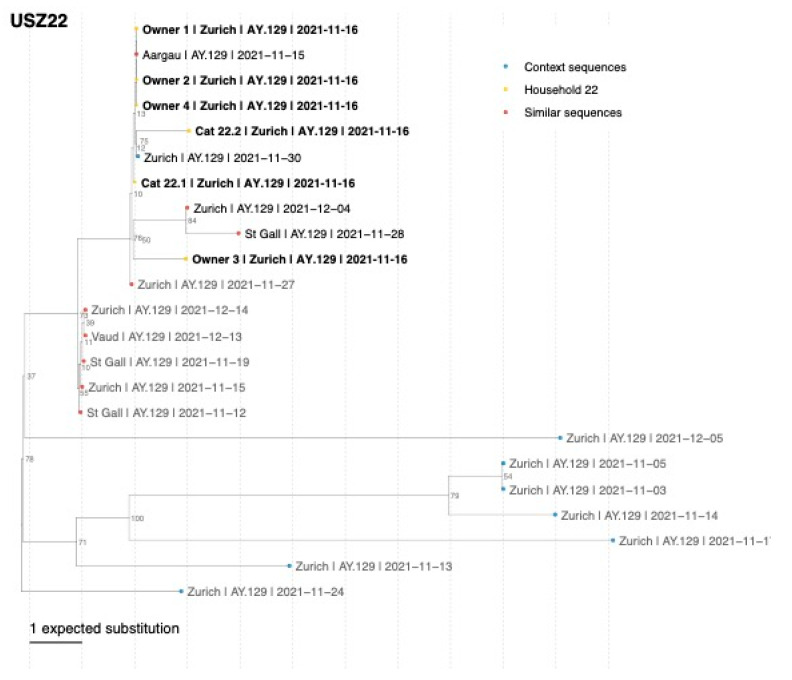
Maximum-likelihood phylogenetic tree showing the relationship between the six sequences of USZ22 (yellow dots and bold text) and their respective 10 most genetically similar Swiss sequences obtained from GISAID. The most similar sequences (red dots) are defined as those with the smaller number of SNPs differences to each household sequence (sequences that are part of the 10 closest sequences for several of the household sequences only appear once in the tree). Blue dots indicate a representative set of 10 sequences circulating in Zurich in November 2021 from pangolin lineage AY.129 (context sequences). Location, pangolin lineage, and collection date are shown for each sequence. Bootstrap values for the internal nodes are shown next to each node from 10,000 bootstrap replicates. Branch length in the tree represents evolutionary distance, i.e., substitutions per site. Reference sequence Wuhan/Hu-1/2019 is used as an outgroup to root the tree.

The SARS-CoV-2 single-nucleotide polymorphisms (SNPs) in the sequences of the household in comparison with the SNPs present in more than 75% of other Swiss sequences of the same pangolin lineage of the Delta variant and the other sequences within the household are presented in Figure 4A. With respect to the other Swiss sequences of lineage AY.129 submitted to GISAID, five SNPs that are rare for the lineage (detected in less than 75% of the AY.129 Swiss sequences) were present in at least one of the sequences of household USZ22. From these five SNPs, two non-synonymous SNPs in ORF1a gen and one in ORF3a gen are very rare (shared by less than 5% GISAID sequences of the lineage in calendar week 45). SNP A10323G (ORF1a:K3353R) was found in every owner and companion animal of household USZ22. Mutation C25463T (ORF3a:T24I) was present in the viral sequence from the cat (22.2) only and less than 1% of AY.129 sequences. The viral sequence from dog 22.1 was identical to the viral sequences of owners one, two, and four. We identified one SNP between the animals’ viral sequences (dog 22.1 and cat 22.2) and one SNP between cat 22.2 and the most similar sequences from owners one, two, and four in the household (Table 7). A phylogenetic tree with the 10 most genetically similar Swiss sequences to each of the sequences from household USZ22 is presented in Figure A2. While the sequences obtained from the household cluster together, they were also very closely related to other viral sequences from the cantons of Zurich, St. Gallen, and Aargau.

**Figure A3:**
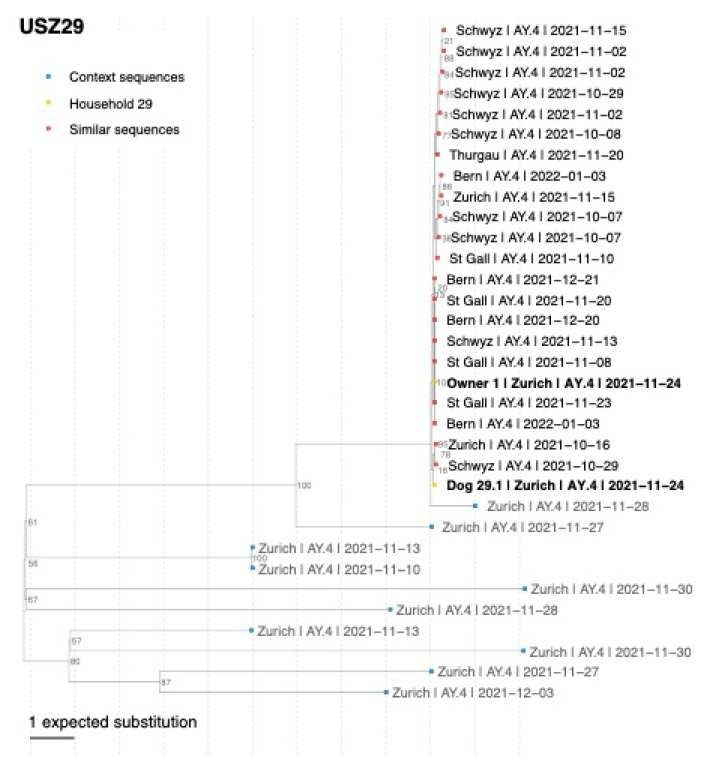
Maximum-likelihood phylogenetic tree showing the relationship between the two sequences of USZ29 (yellow dots and bold text) and their respective 10 most genetically similar Swiss sequences obtained from GISAID. The most similar sequences (red dots) are defined as those with the smaller number of SNPs differences to each household sequence (sequences that are part of the 10 closest sequences for several of the household sequences only appear once in the tree). Blue dots indicate a representative set of 10 sequences circulating in Zurich in November–December 2021 from pangolin lineage AY.4 (context sequences). Location, pangolin lineage, and collection date are shown for each sequence. Bootstrap values for the internal nodes are shown next to each node from 10,000 bootstrap replicates.

In household USZ29, we identified the SARS-CoV-2 Delta-variant pangolin lineage AY.4 (Nextclade clade 21J) in dog 29.1 and owner 1. AY.4 (alias of B.167.2.4) was a lineage with a wide distribution in the United Kingdom. The proportion of AY.4 in the canton of Zurich in the calendar week 47 was 17.5% [53,59]. In comparison to other AY.4 Swiss sequences, 10 rare SNPs (shared by less than 75% of all AY.4 Swiss sequences in GISAID) are present in the household sequences. None of them are very rare mutations for lineage AY.4 (all SNPs in Figure 4B are shared by >10% of AY.4 sequences). The viral sequences from dog 29.1 and owner 1 in household USZ29 were genetically identical without considering the missing positions in the sequences (Figure 4B and Table 7). In the phylogenetic analysis, the viral sequences of household USZ29 cluster with other viral sequences from the canton of Zurich collected at the time the household was sampled (end of November 2021) and with sequences from cantons Schwyz and St. Gallen collected in October and November 2021 (Figure A3).

**Figure A4:**
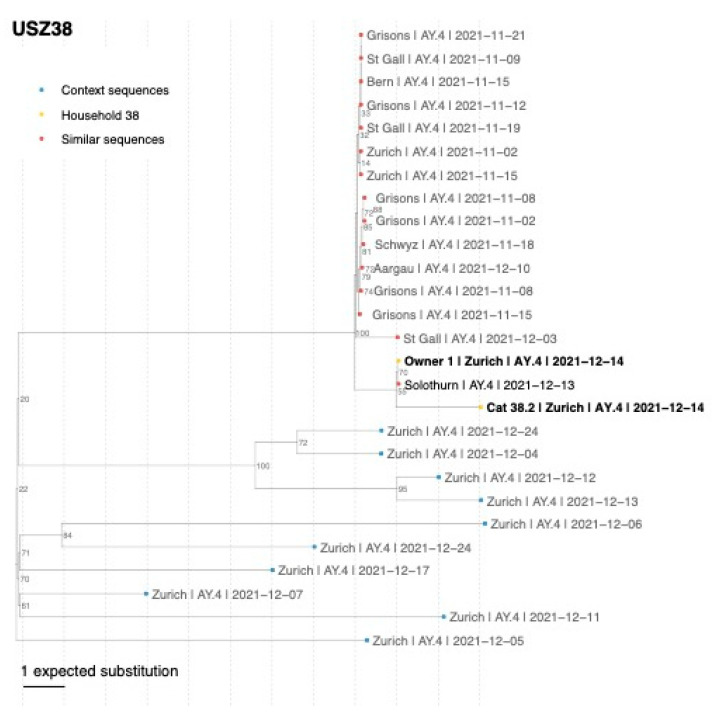
Maximum-likelihood phylogenetic tree showing the relationship between the three sequences of USZ38 (yellow dots and bold text) and their respective 10 most genetically similar Swiss sequences from GISAID. The most similar sequences (red dots) are defined as those with the smaller number of SNPs differences to each household sequence (sequences that are part of the 10 closest sequences for several of the household sequences only appear once in the tree). Blue dots indicate a representative set of 10 sequences circulating in Zurich in November–December 2021 from pangolin lineage AY.4 (context sequences). Location, pangolin lineage, and collection date are shown for each sequence. Bootstrap values for the internal nodes are shown next to each node from 10,000 bootstrap replicates.

In the second household, USZ38, we identified the Delta-variant pangolin lineage AY.4 (Nextclade clade 21J). This variant was observed in the positive-tested cat 38.2 and owner 1 (Table 6). The proportion in the canton of Zurich in the calendar week 50 was 9% of all the sequenced samples [53,59]. Nine SNPs in the cat viral sequence were identified that were very rare, i.e., shared by less than 5% of AY.4 sequences in GISAID the week of sampling, five synonymous and four non-synonymous, in genes ORF1b and ORF7a. Three SNPs between the viral sequences from cat 38.1 and owner 1 were identified (Table 7 and Figure 4C). In the phylogenetic analysis, the sequences from the household USZ38 cluster together with genetically similar sequences observed in Swiss cantons Solothurn, Grisons, St. Gallen, and Zurich at the time when the household tested positive (Figure A4).

**Figure A5:**
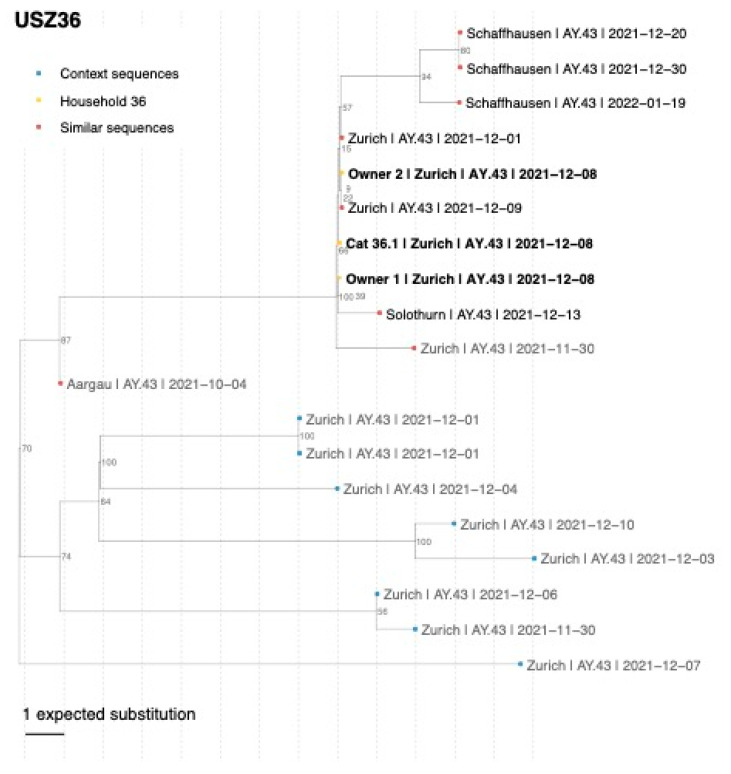
Maximum-likelihood phylogenetic tree showing the relationship between the three sequences of USZ36 (yellow dots and bold text) and their respective 10 most genetically similar Swiss sequences from GISAID. The most similar sequences (red dots) are defined as those with the smaller number of SNPs differences to each household sequence (sequences that are part of the 10 closest sequences for several of the household sequences only appear once in the tree). Blue dots indicate a representative set of 10 sequences circulating in Zurich in December 2021 from pangolin lineage AY.43 (context sequences). Location, pangolin lineage, and collection date are shown for each sequence. Bootstrap values for the internal nodes are shown next to each node from 10,000 bootstrap replicates.

In household USZ36, the sequences were classified as pangolin lineage AY.43 (Nextclade clade 21J). The proportion of AY.43 in the canton of Zurich in calendar week 49 was 16% of all the sequenced samples [53,59]. In comparison with AY.43 Swiss sequences, nine SNPs present in the household sequences were found in <75% of AY.43 sequences (Figure 4D). From these nine SNPs, eight of them were present in all sequences of the household and seven SNPs were particular to the household (<5% of all AY.43 sequences in GISAID the week of sampling). From these mutations, three were non-synonymous and were in the ORF1b and ORF1b genes. The viral sequences obtained from cat 36.1 and owners 1 and 2 are genetically identical (Table 7a). The sequences of the household USZ36 cluster in the phylogenetic tree together with a group of seven similar sequences from Zurich, Schaffhausen, and Solothurn (Figure A5).

**Figure A6:**
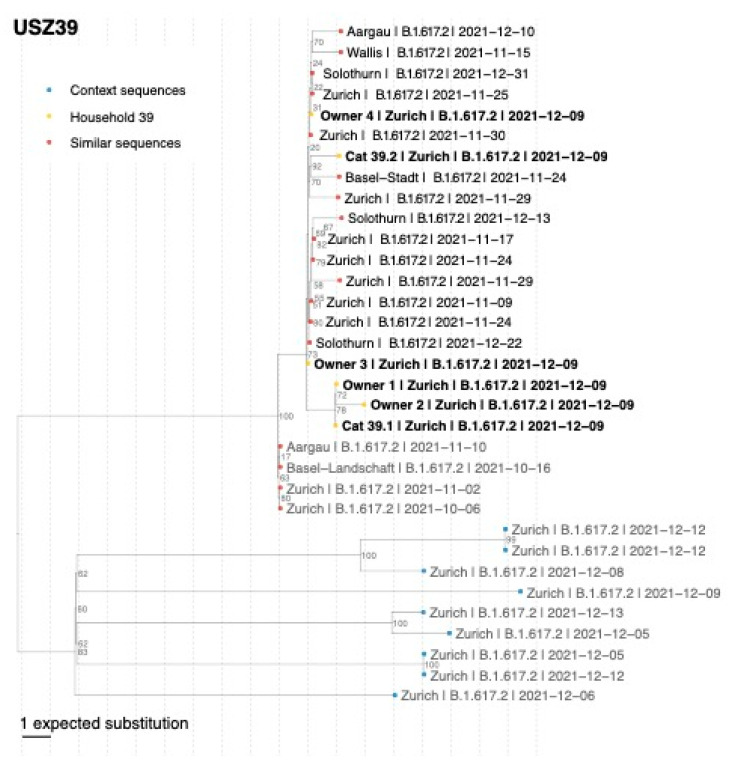
Maximum-likelihood phylogenetic tree showing the relationship between the five sequences of USZ39 (yellow dots and bold text) and their respective 10 most genetically similar Swiss sequences from GISAID. The most similar sequences (red dots) are defined as those with the smaller number of SNPs differences to each household sequence (sequences that are part of the 10 closest sequences for several of the household sequences only appear once in the tree). Blue dots indicate a representative set of 10 sequences circulating in Zurich in November–December 2021 from pangolin lineage AY.43 (context sequences). Location, pangolin lineage, and collection date are shown for each sequence. Bootstrap values for the internal nodes are shown next to each node from 10,000 bootstrap replicates.

In household USZ39, SARS-CoV-2 Delta-variant pangolin lineage B.1.617.2 (Nextclade clade 21J) in cats 39.1 and 39.2 as well as in the four infected owners using NGS was identified. The distribution in owners of pangolin lineage B.1.617.2 in the canton of Zurich in calendar week 49 was 1% of all the sequenced samples [53,59]. An overview of the 16 rare SNPs in any of the household sequences with respect to the common mutations in the Delta variant (shared by more of 75% of B.1.617.2 Swiss sequences in GISAID) is presented in Figure 4E. Only two SNPs in the household sequences were shared by less than 5% of AY.43 sequences, C7390T in owner 2, and non-synonymous mutation C27476T (ORF7a:T28I) indicated with a red box in Figure 4E in cat 39.1 and owners 1 and 2. There were two SNPs between the viral sequences of the two cats, and zero respective SNPs from cats 39.1 and 39.2 in relation to the most similar sequence from an owner (owner 1 and 4, respectively) in the household (Table 7). In the phylogenetic tree, the sequences from cat 39.1 and owners 1 and 2 cluster together in the tree, as well as those from cat 39.2 and owners 3 and 4 (Figure A6). The sequence from owners three and four is ancestral to the other household sequences and identical to other Zurich and Solothurn B.1.617.2 sequences. The most similar sequences were mainly obtained from the canton of Zurich in November and from other nearby cantons in December 2021.

In the animal sequences of the current study, a total of 44 rare SNPs, i.e., shared by less than 75% of the viral sequences of the same lineage in GISAID, were present in at least one of the viral sequences recovered from the animals of the study, and ten of them were very rare, i.e., shared by less than 5% of the viral sequences of the lineage in GISAID. From the 44 SNPs, 25 were synonymous and 19 were non-synonymous substitutions. There were six non-synonymous substitutions in ORF1a, six in ORF1b, two in ORF3a, two in ORF7a, one in ORF8, and two in the spike protein. In the case of the ten very rare mutations (shared by less than 5% of the same lineage), all of them were non-synonymous mutations in ORF1a, ORF1b, ORF3a, or ORF7a. The median number of rare mutations in the viral sequences from the study animals was nine SNPs, similar to the number of rare mutations in all Swiss sequences of the same lineages submitted to GISAID (median 8, 0.25 quantile 5, and 0.75 quantile 11 SNPs). Four SNPs were exclusively present in the viral sequences obtained from the animals and not from the owners of the household: SNP C25463T (ORF3a:T24I) in cat 22.2, A21194G (ORF1b:D2576G) and T29867A in cat 38.2, and A21743C in cat 39.2. In comparison to the other viral sequences from companion animals in other countries, 12 out of the 44 rare SNPs are shared by the animal viral sequences obtained from this study and other companion animal (dogs and cats) viral sequences. SNP A10323G (ORF1a:K3353R) was shared by dog 22.1 and cat 22.2 and six other animal sequences from GISAID (four cats and one dog from the USA, EPI_ISL_3010054, EPI_ISL_3128559, EPI_ISL_10656104, EPI_ISL_3010053, and EPI_ISL_3148880, and one dog from Colombia: EPI_ISL_8422346), present in 2.62% of all companion animal sequences (95% confidence interval 1.28–5.31%), while it is shared by only 1.41% of all human viral sequences in GISAID. In addition, A10323G is not of any specific lineage or variant in particular. One spike mutation, G25244T (S:V1228L), was present in dog 29.1, in two animal sequences from other countries (1.12% of all companion animal sequences, 95% confidence interval 0.38–3.25%) and 0.12% of all SARS-CoV-2 sequences from human samples. There were ten other mutations in the study shared by animal sequences in GISAID and the study sequences; eight of them with a lower share of total sequences in GISAID are described in Table A1.

## 4. Discussion

This study describes the first confirmed cases of VOC Delta B.1.617.2 in companion animals in Switzerland. Pangolin lineages AY.129 and AY.4 could be described in animals for the first time worldwide. All cats and dogs lived in COVID-19-affected households. Eight cats and two dogs living in these households showed positive RT-qPCR results in either nasal, oral, or fecal swabs. For three dogs and nine cats, infection was confirmed by the presence of anti-SARS-CoV-2 antibodies and/or neutralizing activity. In most households, direct transmission between owners and animals is most likely, while in the multi-pet households (USZ39, USZ22), animal-to-animal transmission could not be excluded.

The SARS-CoV-2 sequences of owners and their animals within a household in our study were genetically similar and belonged to the same pangolin lineage as determined via NGS. In general, a high genetic similarity of SARS-CoV-2 sequences within a population is indicative of epidemiological linkage, which in turn can be suggestive of direct transmission among individuals or indirect transmission via surfaces. Based on the genetic sequences of SARS-CoV-2 in GISAID, we did not find evidence of a dominant Delta sublineage in companion animals in general and also not in the current study. Viral sequences identified in the companion animals of the current study showed between zero and three SNP differences from the most similar sequence in their owner(s). Our results are consistent with those of the previous studies of SARS-CoV-2 infection in companion animals and their owners, which reported very similar sequences with up to 99.9% nucleotide identity of animals and owners or people in the close geographic area [60,61,62]. A genomic mutation rate of 2.9 × 10^−6^ nt^−1^ cycle^−1^ was experimentally described [63] (approximately one mutation for each two new infections). In humans, direct transmission has been considered likely when fewer than two SNPs [64] or less than one SNP [65] difference were present. In our study, we observed such low SNP differences within households, indicative of within-household transmission, except for household USZ38. Specifically, the sequences obtained from the animals were identical to the sequences from one of the owners of the household in four out of overall seven cases with the three exceptions being cats 22.1 (one SNP difference), 39.2 (one SNP), and 38.2 (three SNPs). In the latter case (household USZ38), three SNP differences between owner and cat were detected, possibly indicating different origins of the infection in the individuals. However, one cannot prove direct transmission based only on SNP differences in the consensus sequence and, furthermore, raising hypotheses is difficult due to the low diversity in SARS-CoV-2 sequences [66]. However, combining SNP data with epidemiological data allows us to investigate plausible transmission histories [67]. In our study, we performed this analysis for each household, thereby strongly suspecting within-household transmission between owners and animals in most cases due to the clustering of household sequences, relatively low number of SNP differences and the known exposure to SARS-CoV-2. Infection in some cases was associated with reported close contact to the animals, e.g., in USZ29, the two animals (dog 29.1 and cat 29.3) with which the infected children had very close contact became infected; the other two cats, that were in less intense contact, (29.2 and 29.4) tested negative. This indicates that environmental contamination with SARS-CoV-2 in COVID-19 households alone might not be the main driver for the infection of the companion animals living there. Overall, our data are suggestive of at least six suspected within-household transmission events between owner and animals as indicated by two or fewer SNP differences between the viral animal and owner sequences and reported direct contact between the owner and animal. This included the following households and owner–animal pairs: in household USZ22, owners 1, 2, and 4–dog 22.1 and cat 22.2; in household USZ29, owner 1–dog 29.1; in household USZ39, owner 1–cat 39.1 and owners 3 and 4–cat 39.2; and in household USZ36, owners 1 and 2–cat 36.1.

On the other hand, animal-to-animal transmission can be considered if more than one pet in a multi-pet household is infected. Animal-to-animal transmission has experimentally been reported previously in ferrets [68,69,70], cats [70,71,72,73], hamsters [74], and fruit bats [75]. In two multi-pet households in the current study (USZ22 and USZ39), several animals were SARS-CoV-2-positive and sequencing results were available for all animals. In household USZ22, animal-to-animal transmission of the SARS-CoV-2 Delta variant cannot be ruled out, given only one SNP difference between dog 22.1 and cat 22.2. However, other routes are also plausible: the dog 22.1 sequence was identical to the sequence from one of the owners; therefore, owner-to-animal transmission was also plausible for this dog. The sequence from cat 22.2 had only one SNP difference compared to the sequences from three owners; therefore, for cat 22.2, animal-to-animal transmission and transmission between owner and animal were plausible. In household USZ39, from which viral sequences from two cats were available, the animal sequences were separated by only two SNPs. However, the animals’ sequences were more closely related to the owner sequences (identical for cat 39.1 and owner 1, and one SNP difference for cat 39.2 and owners 3 and 4); therefore, also in this household, transmission between owner and animal is a plausible transmission history. In households USZ22, USZ49, and USZ211, the cats had no outdoor access so that an infection route other than by the owners, or in the cases of USZ211 and USZ49 by dog 22.1, respectively, or the second cat, can almost be ruled out, meaning for these households the data suggest a transmission direction from owners to animals. A different source of transmission not included in the study even though the intra-household transmission was to be regarded as strongly probable, especially in the temporal context to the infection of the owners, cannot be excluded.

Four particular SNPs were exclusively present in the viral sequences obtained from the animals and not from the owners in the households of the present study, and there were 12 mutations in the animals of the current study that were shared with animal sequences from GISAID. The frequencies of these shared mutations, which were partly higher than in the human sequences, could indicate that they were over-represented in the companion animal population and some of these mutations may have some selective advantage that suggested an adaptation to animal hosts. However, we highlight that we merely describe empirical patterns and thus potential candidate mutations which may have a selective advantage, while not testing for significance. For example, three SNPs were also exclusively present in humans but not the accompanying animals. Nevertheless, previous experimental results have also indicated that viral adaptation to host animals could increase variant selection [76,77] and reinforces the need for a One Health approach in monitoring SARS-CoV-2 in animals to prevent the development of animal reservoirs.

In the present study, viral RNA loads were determined. We also attempted virus isolation, but the approach was mainly hampered by biosafety and logistical issues, which resulted in the inability to rapidly obtain native swab samples from COVID-19-affected households and at timepoints when virus loads were high. Some animal samples had RT-qPCR Ct values < 30, which may be indicative of a virus load associated with significantly higher infectivity than samples with a higher Ct value: Ct values > 30 were usually associated with decreased infectivity [78]. Therefore, we were left to speculate that at least some of the infected companion animals in the COVID-19 households presented in this study were shedding infectious viruses at some timepoints.

RT-qPCR results obtained from different swabs (nasal, oral, and fecal) from one animal do not always show corresponding results; in four animals, the nasal swabs tested positive over a longer time period than the oral swabs, while in five animals it was vice versa. Thus, our data indicate that no single sample material is optimal to detect all infected cats and dogs. Intermittent positivity was observed in some households over the sampling period. This was especially notable in oral swabs. Previous studies have shown intermittent shedding in various sample materials in up to 38% of human patients [79,80,81], and intermittent shedding has also been previously described in animals [82]. The possible reasons for intermittent shedding were presumably a weak or temporarily suppressed inflammatory response, which then allowed the reactivation of the virus [83]. Intermittent positivity can also occur due to Ct values which are at the detection limit [84] and CT values, in the cases where intermittent shedding occurred in animals of our current study, had a Ct value at least >30. The longest observed shedding period of RNA in the present study was recognized in a cat; it was 16 days. The maximal shedding period within our study may indeed have been even longer since the duration and frequency of sampling varied depending on the household. Nevertheless, with an average of 4.7 swab sample collections per household in an average surveillance period of 18.5 days, we were able to cover a wide range of the infection period with close monitoring.

For the positive-tested surface samples in our study, it was impossible to determine the source of viral RNA; it could have resulted from viral shedding from the animal or contamination by the owner [6]. Remarkably, in our study, positive fur and bed samples were also collected from cats that tested negative on oral, nasal, and fecal swabs (cats 38.1, 29.2, and 29.4). Thus, viral shedding caused by these animals seems rather unlikely as the source of fur and bed contaminations. Rather, the animals’ fur and bedding could have been contaminated by their infected owners or the other shedding animals in the household through direct contact, e.g., during petting or grooming, or via indirect contact. Intermittent positive results were also observed for some of the surface samples. However, intermittent positivity of surface samples could also have occurred due to the variable hygiene management of the household or changes in the behavior of the animals (e.g., washing of the animal’s bed or grooming behavior of cats). Nevertheless, since in another study samples obtained from surfaces with a Ct > 30 (low RNA loads) did not yield replicating viruses as determined by cell culture assays [85], the majority of the environmental samples in our study were assumed to be non-infectious. Viral titers, which were below a Ct value of 30 in both assays, and thus, according to the above-mentioned study, could contain infectious viral material, could only be detected in one of the fur samples and eight of the bed samples. Due to logistical and biosafety reasons, virus isolation was not possible, so no titers could be given.

The time interval between the first positive RT-qPCR test in an animal and the blood collection for serology from the same animal ranged between 26 and 102 days. In another study, it was shown that antibodies remained stable or even increased in the first 3 months after infection [86]; taking this information into consideration, it can be suggested that an ideal timepoint for antibody testing should have been available for the majority of the animals in our study. In fact, all animals tested for antibodies within our study were seropositive. Moreover, all animals’ results obtained from the antibody-binding (RBD ELISA) and the neutralization (sVNT) assays were congruent, suggesting that the detected antibodies also had a neutralizing character. One sample obtained from a dog (22.1) collected more than three months after the RT-qPCR-positive result in this animal tested positive in the PVNT, but negative in RBD ELISA and sVNT. The negative RBD ELISA result in this animal could have been due to the antigen used in the ELISA, which was based on the ancestral variant. A study showed that the serum of Delta-variant-recovered patients had a lower reactivity in the RBD with a B.1 strain [87] so that if the sample was only weakly positive, the RBD ELISA result could possibly be negative. Furthermore, according to a comparative study, the performance of sVNT and PVNT was also well—but not completely—correlated [88]. Further investigations due to this result are ongoing.

## 5. Conclusions

In conclusion, four different pangolin lineages of the SARS-CoV-2 Delta variant were detected in the present study using RT-qPCR and confirmed via sequencing. This included two lineages that have not previously been described in animals (AY.4 and AY.129). The infections with the SARS-CoV-2 Delta variant were confirmed serologically in 12 companion animals. Some infected animals showed respiratory or gastrointestinal signs coinciding with SARS-CoV-2 Delta variant infections. All animals lived in COVID-19-affected households and in close contact with SARS-CoV-2-infected owners. Thus, direct transmission was assumed to be the main source of the infection in the studied households. This assumption was also plausible when considering the sequencing data with a low number of SNP differences between viral sequences of animals and their owners. Nevertheless, in the two multi-pet households, from which sequences were available, animal-to-animal transmission could not be excluded based on the sequencing results. Our NGS data identified shared SNPs between the viral sequences of our animals and other publicly available companion animals, as well as SNPs that were exclusively observed in the animals of our study but not in the owners. It remains to be investigated if these mutations are involved in the adaptation of the virus to the animal host or occurred merely by chance; of note, adaptation has already been described experimentally. The results of our study support and underline the importance of monitoring animals in close contact with SARS-CoV-2-infected humans in a One Health context.

## Figures and Tables

**Table 1:** Overview of COVID-19-affected households, date of first RT-qPCR test results, number of infected owners and animals, and their RT-qPCR and serological results.

Household			Owners		Dogs			Cats		
ID	Date Entering Study	Canton	People in Household	RT-qPCR Positive ^1^	ID	RT-qPCR Positive	Sero-Positive	ID	RT-qPCR Positive	Seropositive
USZ22	11 November 2021	ZH	2 adults, 3 children	2 adults, 2 children	Dog 22.1	**Yes**	**Yes ^2^**	Cat 22.2	**Yes**	**Yes**
USZ29	21 November 2021	ZH	2 adults, 2 children	1 adult, 2 children	Dog 29.1	**Yes**	**Yes**	Cat 29.2 Cat 29.3 Cat 29.4	No **Yes** No	N/A N/A N/A
USZ39	2 December 2021	ZH	2 adults, 2 children	2 adults, 2 children				Cat 39.1 Cat 39.2	**Yes** **Yes**	**Yes** **Yes**
USZ38	4 December 2021	ZH	1 adult, 2 children	2 children				Cat 38.1 Cat 38.2	No **Yes**	N/A N/A
USZ36	5 December 2021	ZH	2 adults, 2 children	2 adults				Cat 36.1	**Yes**	**Yes**
USZ174	11 December 2021	ZH	2 adults	2 adults				Cat 174.1 ^3^ Cat 174.2 ^3^	N/A N/A	**Yes** **Yes**
USZ49	19 December 2021	ZH	1 adult	1 adult				Cat 49.1 Cat 49.2	**Yes ^4^** **Yes**	**Yes ^5^** **Yes**
USZ149	21 December 2021	SG	≥1 ^6,7,8^	At least 1	Dog 149.1 ^3^	N/A	**Yes**			
USZ211	9 February 2022	SG	≥1 ^6,7^	At least 1				Cat 211.1 ^3^	N/A	**Yes**
Total						2/2	3/3		8/11	9/9

^1^ Results also partly obtained from commercial laboratories; ^2^ slightly neutralizing titer in PVNT for Delta variant, negative in ELISA and sVNT; ^3^ serologically confirmed animals, no swab samples available, ^4^ Cat 49.1 confirmed as questionably positive in RT-qPCR (Ct value > 38 and <45); ^5^ in ELISA and PVNT, not in sVNT; ^6^ no swabs from owners available for this study; RT-PCR result from commercial laboratory; ^7^ at least one person in the household, no further information available; ^8^ animal care taker in kennel where dog was kept; N/A: not available. Bold = positive results.

**Table 2:** Characteristic of all cats living in the SARS-CoV-2 Delta-lineage-positive households and clinical signs observed.

Cat ID	SARS-CoV-2 Positivity	Age (Years)	Sex	Breed	Outdoor Access	Pre-Existing Condition	Clinical Signs	Clinical Signs Observed ^1^
Cat 22.2	RNA, Abs	unknown	fn	Oriental Shorthair	No		No	
Cat 29.3	RNA	5	f	Europ. Shorthair	Yes		No	
**Cat 39.1** Cat 39.2	RNA, Abs RNA, Abs	8 13	mn fn	Unknown Unknown	Yes Yes	Arthrosis	Yes No	Respiratory (nasal discharge)
**Cat 38.1** **Cat 38.2**	No RNA	1.5 1.5	mn mn	Bengal Bengal		Giardia infection Giardia infection	Yes Yes	Respiratory and gastrointestinal (ocular discharge, vomiting) Respiratory and gastrointestinal (ocular discharge, vomiting); lethargy, apathy, reduced appetite, bacterial cystitis ^2^
Cat 36.1	RNA	18	fn	Mixed breed		Hyper-thyreosis	No	
Cat 49.1 Cat 49.2	RNA, Abs RNA, Abs	11 17	mn mn	Somali Maine Coon	No No		No No	
Cat 174.1 Cat 174.2	Abs Abs	9 9	mn mn	Europ. Shorthair Europ. Shorthair	Yes Yes		No No	
**Cat 211.1**	Abs	10	f	Europ. Shorthair	No		Yes	Gastrointestinal ^2^ (vomiting, absent defecation)

^1^ If not indicated otherwise, clinical signs were observed and reported by the animal owners. ^2^ Clinical signs observed by private veterinary practitioner; f: female; fn: female neutered; m: male; mn: male neutered; Abs: antibodies; RNA: viral ribonucleic acid detected using RT-qPCR; bold: clinical signs present.

**Table 3:** Characteristic of SARS-CoV-2 Delta-lineage-positive dogs and clinical signs observed.

Dog ID	SARS-CoV-2 Positivity	Age (Years)	Sex	Breed	Pre-Existing Condition	Clinical Signs	Clinical Signs Observed ^1^
Dog 22.1	RNA, Abs	unknown	mn	Australian Labradoodle		No	
**Dog 29.1**	RNA, Abs	1	f	Labrador–Bernese Mountain Mix		Yes	Lethargy/apathy
**Dog 149.1**	Abs	9	f	Rhodesian Ridgeback	Mammary tumor, pyometra	Yes	Respiratory (sneezing) ^2^

^1^ If not indicated otherwise, clinical signs were observed and reported by the animal owners. ^2^ Clinical signs observed by private veterinary practitioner; f: female; mn: male neutered; Abs: antibodies; RNA: viral ribonucleic acid detected using RT-qPCR; bold: clinical signs present.

**Table 4:** Enzyme-linked immunosorbent assay that detects antibodies binding to the SARS-CoV-2 spike glycoprotein receptor-binding domain (RBD ELISA) and SARS-CoV-2 Surrogate Virus Neutralization Test Kit (sVNT) results in cats and dogs in COVID-19-affected households.

Animal ID ^1^	Date of Blood Collection for Serology	Days after First Positive RT-qPCR of the Animal	RBD Elisa	Percentage of Positive Control	sVNT	Percentage Inhibition
Dog 22.1	28 February 2022	102	Negative		Negative	
Cat 22.2	28 February 2022	100	Positive	71	Positive	88
Dog 29.1	28 December 2021	34	Positive	33	Positive	62
Cat 39.1	3 February 2022	56	Positive	151	Positive	100
Cat 39.2	3 February 2022	56	Positive	125	Positive	95
Cat 36.1	3 January 2022	26	Positive	130	Positive	94
Cat 49.1	8 March 2022	69	Positive	102	Positive	100
Cat 49.2	8 February 2022	41	Positive	96	Negative ^2^	69
Dog 149.1	21 December 2022	N/A ^3^	Positive	55	Positive	58
Cat 174.1	21 December 2022	N/A ^3^	Positive	125	Positive	100
Cat 174.2	21 December 2022	N/A ^3^	Positive	135	Positive	100
Cat 211.1	9 February 2022	N/A ^3^	Positive	147	Positive	101

^1^ Only animals are listed with samples available for serology (see also Table 1). ^2^ Not positive in sVNT, but confirmed serologically positive in PVNT ^3^. N/A: not available: no swab samples and thus no RT-qPCR result available for these animals.

**Table 5:** Pseudotype-based neutralization assay (PVNT) results: variant-specific antibodies for Alpha, Beta, Delta, or Omicron variants.

Animal ID	Pseudotype-Based Neutralization Assay (Neutralization Titers ^1^)				Identified Variant
	Alpha	Beta	Delta	Omicron	
Dog 22.1	<50	<50	**58**	<17	Delta
Cat 22.2	<50	<50	**115**	20	Delta
Dog 29.1	<50	55	**94**	<17	Delta
Cat 39.1	68	71	**166**	27	Delta
Cat 39.2	143	101	**224**	25	Delta
Cat 36.1	49	<50	**463**	22	Delta
Cat 49.1	91	72	**229**	<50	Delta
Cat 49.2	<50	<50	**74**	<50	Delta
Dog 149.1	<50	<50	**261**	<17	Delta
Cat 174.1	225	276	**550**	74	Delta
Cat 174.2	411	707	**1890**	379	Delta
Cat 211.1	240	254	**341**	52	Delta

^1^ Samples with no measurable titers are considered negative. For samples with one or more titers, the pseudotype variant that generates the highest titer indicates the variant the animal has likely been infected with (bold types).

**Table 6:** Results from sequencing of samples from RT-qPCR-positive individuals: variant and pangolin lineage analyses.

Household ID	Sample from	Date of Sampling for Sequencing	Material of Sequenced Sample	Breadth of Sequencing Coverage	Depth of Sequencing Coverage	SARS-CoV-2 Variant	Pangolin Lineage
USZ22	Owner 1 Owner 2 Owner 3 Owner 4 Dog 22.1 Cat 22.2	16 November 2021 16 November 2021 16 November 2021 16 November 2021 18 November 2021 16 November 2021	Nasal swab Oral swab Nasal swab Nasal swab Oral swab Oral swab	99.8% 99.8% 99.8% 99.8% 99.8% 99.0%	10,078.325 7357.6606 5991.9077 8399.461 7603.86 8748.53	Delta Delta Delta Delta Delta Delta	AY.129 AY.129 AY.129 AY.129 AY.129 AY.129
USZ29	Owner 1 Dog 29.1 Cat 29.3	24 November 2021 24 November 2021 24 November 2021	Nasal swab Nasal swab Oral swab	99.8% 99.4% 71.3%	7085.115 8431.732 16,659.176	Delta Delta -	AY.4 AY.4 -
USZ39	Owner 1 Owner 2 Owner 3 Owner 4 Cat 39.1 Cat 39.2	9 December 2021 9 December 2021 9 December 2021 9 December 2021 9 December 2021 9 December 2021	Nasal swab Nasal swab Oral swab Oral swab Oral swab Oral swab	99.7% 99.8% 99.7% 99.9% 99.7% 98.8%	21,950.77 20,430.922 303.0392 14,888.675 19,432.705 22,502.582	Delta Delta Delta Delta Delta Delta	B.1.617.2 B.1.617.2 B.1.617.2 B.1.617.2 B.1.617.2 B.1.617.2
USZ38	Owner 1 Owner 2 Cat 38.2	14 December 2021 14 December 2021 14 December 2021	Nasal swab Nasal swab Nasal swab	98.8% 90.3% 98.7%	15,422.453 13,471.375 16,428.799	Delta - Delta	AY.4 - AY.4
USZ36	Owner 1 Owner 2 Cat 36.1	8 December 2021 8 December 2021 8 December 2021	Nasal swab Nasal swab Oral swab	99.8% 99.8% 99.1%	18,054.1 17,920.172 18,757.414	Delta Delta Delta	AY.43 AY.43 AY.43

**Table 7:** Overview of the SNP differences between animals and owners within the household, animals within the household as well as in the other households, and animals and other human sequences in GISAID. Pairwise distance is measured as the number of sites that differ between the two sequences. Missing positions are ignored.

Household ID	Sample from	Minimum Distance (SNPs) to Sequence from Animal in the Household	Minimum Distance (SNPs) to Sequence from Owner in the Household	Most Similar Sequence from Owner in the Household	Minimum Distance (SNPs) to Sequence from Animal in Another Household	Most Similar Sequence from Animal in Another Household	Minimum Distance (SNPs) to Another Swiss Sequence
USZ22	Dog 22.1 Cat 22.2	1 1	0 1	Owners 1, 2, 4 Owners 1, 2, 4	13 14	Cat 38.2 Cat 38.2	0 1
USZ29	Dog 29.1	-	0	Owner 1	15	Dog 22.1	0
USZ39	Cat 39.1 Cat 39.2	2 2	0 1	Owner 1 Owners 3, 4	18 17	Dog 22.1 and cat 22.2 Dog 22.1 and cat 22.2	1 1
USZ38	Cat 38.2	-	3	Owner 1	13	Dog 22.1	2
USZ36	Cat 36.1	-	0	Owners 1, 2	16	Dog 22.1 and cat 22.2	0

## Data Availability

RT-qPCR confirmed animals acquired in this study have been deposited in WOAH under accession numbers: ob_93511-Oerlikon (22D1, 22C2), ob_94514-Dübendorf (39C1, 39C2), ob_94511-Winterthur (36C1), ob_94513-Uerikon (38C1, 38C2), ob_94197-Hombrechtikon (29D1, 29C3), ob_95431-Zumikon (49C2). NGS viral genetic sequences have been submitted to GISAID with accession numbers: EPI_ISL_9461296, EPI_ISL_9461294, EPI_ISL_9461297, EPI_ISL_9461299, EPI_ISL_9461301, EPI_ISL_9461298 (22C2, 22D1, and the owners 1–4 viral sequences from household USZ22), EPI_ISL_9461295, EPI_ISL_9461300 (29D1, owner 1 from USZ29), EPI_ISL_11583324, EPI_ISL_11583323, EPI_ISL_11583322 (36C1, owners 1–2 USZ36), EPI_ISL_11583327, EPI_ISL_11583326 (38C2, owner 1 USZ38), EPI_ISL_11583331, EPI_ISL_11583332, EPI_ISL_11583328, EPI_ISL_11583329, EPI_ISL_16125540, EPI_ISL_11583330 (39C1, 39C2, owners 1–4 USZ39), and GenBank with accession numbers: ON982614, ON982612, ON982615, ON982617, ON982619, ON982616 (22C2, 22D1, and owners 1–4 viral sequences from household USZ22), ON982613, ON982618 (29D1, owner 1 from USZ29), ON982604, ON982602, ON982603 (36C1, owners 1–2 USZ36), ON982606, ON982605 (38C2, owner 1 USZ38), ON982610, ON982611, ON982607, ON982608, OQ050229, ON982609 (39C1, 39C2, owners 1–4 USZ39).

## Supplementary Materials

The supporting information can be downloaded at: https://www.mdpi.com/article/10.3390/v15010245/s1.

## Appendix A

**Table A1:** Rare and very rare, shared SNPs between companion animal sequences of this study and other companion animal sequences in GISAID. The percentage in which the SNP is found in all human, animal, and companion animal sequences in GISAID is calculated together with its 95% binomial proportion confidence interval using the Wilson method. Very rare mutations for the lineages in the study samples are indicated in bold.

	SNP	Aa Mutation	Samples	% Human Sequences GISAID	% Animal Sequences GISAID	% Companion Animal Sequences GISAID
1	**A10323G**	ORF1a:K3353R	22.2, 22.1	1.41 (1.4, 1.42)	0.93 (0.61, 1.42)	2.62 (1.28, 5.31)
2	**A929G**	ORF1a:I222V	36.1	0.06 (0.06, 0.06)	0.13 (0.05, 0.39)	0.75 (0.21, 2.69)
3	C15952A	ORF1b:L829I	39.1, 39.2	2.36 (2.35, 2.36)	0.44 (0.24, 0.81)	0.75 (0.21, 2.69)
4	C20844T	-	36.1	0.17 (0.17, 0.17)	0.88 (0.57, 1.36)	1.12 (0.38, 3.25)
5	C22792T	-	39.1, 39.2	5.66 (5.65, 5.68)	0.97 (0.64, 1.47)	1.12 (0.38, 3.25)
6	C6026T	ORF1a:P1921S	29.1	0.15 (0.15, 0.15)	0.13 (0.05, 0.39)	1.12 (0.38, 3.25)
7	C9286T	-	39.1, 39.2	0.2 (0.2, 0.2)	0.97 (0.64, 1.47)	1.87 (0.8, 4.31)
8	G2272A	-	29.1	0.03 (0.03, 0.04)	0.13 (0.05, 0.39)	0.37 (0.02, 2.09)
9	G25244T	S:V1228L	29.1	0.12 (0.12, 0.12)	0.44 (0.24, 0.81)	1.12 (0.38, 3.25)
10	G487A	-	36.1	0.04 (0.04, 0.04)	0.13 (0.05, 0.39)	0.75 (0.21, 2.69)
11	T27534C	-	38.2	0.03 (0.02, 0.03)	0.35 (0.18, 0.7)	1.5 (0.58, 3.79)
12	T29867A	-	36.1, 38.2, 39.1	0.12 (0.12, 0.13)	0.18 (0.07, 0.45)	1.12 (0.38, 3.25)
