# Supplementary material for: Detection and Molecular Characterization of the SARS-CoV-2 Delta Variant and the Specific Immune Response in Companion Animals in Switzerland
Source: Viruses. 2023 Jan 15;15(1):245. doi: 10.3390/v15010245 (PMC9864232; doi:10.3390/v15010245)
Supplement: Supplementary file 1 [file viruses-15-00245-s001.zip › gisaid_supplemental_table_epi_set_221201wf.pdf]

SUPPLEMENTAL TABLE

Data Availability

GISAID Identifier: EPI_SET_221201wf
doi: 10.55876/gis8.221201wf

All genome sequences and associated metadata in this dataset are published in GISAID's EpiCoV database. To view the contributors of each individual sequence with details such as accession number, Virus name, Collection date, Originating Lab and Submitting Lab and the list of Authors, visit 10.55876/gis8.221201wf

Data Snapshot

- EPI_SET_221201wf is composed of 441 individual genome sequences.
- The collection dates range from 2019-12-31 to 2022-01-19;
- Data were collected in 27 countries and territories;
- All sequences in this dataset are compared relative to hCoV-19/Wuhan/WIV04/2019 (WIV04), the official reference sequence employed by GISAID (EPI_ISL_402124). Learn more at https://gisaid.org/WIV04.
